# Structural Analysis of Neutralizing Epitopes of the SARS-CoV-2 Spike to Guide Therapy and Vaccine Design Strategies

**DOI:** 10.3390/v13010134

**Published:** 2021-01-19

**Authors:** Maxwell T. Finkelstein, Adam G. Mermelstein, Emma Parker Miller, Paul C. Seth, Erik-Stephane D. Stancofski, Daniela Fera

**Affiliations:** Department of Chemistry and Biochemistry, Swarthmore College, Swarthmore, PA 19081, USA; mfinkel1@swarthmore.edu (M.T.F.); amermel1@swarthmore.edu (A.G.M.); emiller6@swarthmore.edu (E.P.M.); pseth1@swarthmore.edu (P.C.S.); estanco1@swarthmore.edu (E.-S.D.S.)

**Keywords:** COVID-19, SARS-CoV-2, coronavirus, spike, neutralizing antibodies, immunogen

## Abstract

Coronavirus research has gained tremendous attention because of the COVID-19 pandemic, caused by the novel severe acute respiratory syndrome coronavirus (nCoV or SARS-CoV-2). In this review, we highlight recent studies that provide atomic-resolution structural details important for the development of monoclonal antibodies (mAbs) that can be used therapeutically and prophylactically and for vaccines against SARS-CoV-2. Structural studies with SARS-CoV-2 neutralizing mAbs have revealed a diverse set of binding modes on the spike’s receptor-binding domain and N-terminal domain and highlight alternative targets on the spike. We consider this structural work together with mAb effects in vivo to suggest correlations between structure and clinical applications. We also place mAbs against severe acute respiratory syndrome (SARS) and Middle East respiratory syndrome (MERS) coronaviruses in the context of the SARS-CoV-2 spike to suggest features that may be desirable to design mAbs or vaccines capable of conferring broad protection.

## 1. Introduction

In the past two decades, there have been three emergences of highly pathogenic coronaviruses in humans. The current pandemic is due to the novel severe acute respiratory syndrome coronavirus 2 (nCoV or SARS-CoV-2), which causes coronavirus disease 2019 (COVID-19) and surfaced in December of 2019 in Wuhan, China. As of 31 December 2020, COVID-19 has been diagnosed in >83.4 million people globally and caused >1.8 million deaths. The COVID-19 pandemic was preceded by a highly pathogenic human coronavirus that emerged in the Middle East in 2012 [1]. Middle East respiratory syndrome coronavirus (MERS-CoV) was responsible for 2494 cases of infection and 858 deaths. In 2003, the human severe acute respiratory syndrome coronavirus (SARS-CoV) emerged in China. SARS-CoV spread to 37 different countries and caused 8098 cases of infection, of which 774 (9%) were fatal. 

Vaccines are crucial to combat a worldwide pandemic but typically take years to develop and distribute. Due to the severity of the current pandemic to humans, vaccines have already been approved in record time, and several other vaccines and treatments are currently undergoing Food and Drug Administration (FDA) approval. Since vaccines are unable to confer immediate protection or treat those who have already been infected, immediate solutions are also needed. The use of convalescent plasma from individuals infected with COVID-19 has shown some promise in improving clinical outcomes and decreasing viral loads [2,3,4,5]. Neutralizing monoclonal antibodies (mAbs) have also become a powerful tool in providing prophylactic and therapeutic protection against emerging viruses [6,7]. Due to the propensity of viruses to mutate and develop resistance mutations, it is important to consider treatments and vaccines that are more universal.

SARS-CoV-2, SARS-CoV, and MERS-CoV are all betacoronaviruses, so they have a similar single-stranded, positive-sense RNA genome structure [8,9]. SARS-CoV-2 and SARS-CoV are more closely related to each other than to MERS-CoV and utilize similar host cell entry mechanisms [10]. The coronavirus spike glycoprotein, S, a trimeric assembly that protrudes from the virion surface, plays a critical role in initiating viral infection. S is responsible for coronavirus attachment to the host cell surface receptor and fusion of viral and host cell membranes [11]. Neutralizing mAbs target S and prevent viral entry into host cells, thereby reducing the severity of primary infection and the onset of disease. Nanobodies or VHHs, which contain only a variable heavy domain, have also shown promise and display certain advantages over mAbs. In particular, VHHs have smaller footprints than antibodies that allow them to interact with hard-to-reach regions of the spike. VHHs can also be expressed in bacteria in high yields, have higher thermal stability and chemostability than antibodies, and can be administered by an inhaler directly to the respiratory tract, the most common site of SARS-CoV-2 infection [12].

In this review, we describe recent insights into the structural conformation of the SARS-CoV-2 spike, particularly as it relates to exposure of mAb epitopes. We highlight the different mAb epitopes on SARS-CoV-2 S based on high-resolution structural data and describe alternate epitopes on SARS-CoV and MERS-CoV spikes as well, which might identify new immunogenic regions on SARS-CoV-2 S or cross-neutralizing mAbs. Using this information, we suggest approaches that could lead to more effective therapies. We also highlight alternate target sites and those that might elicit broader responses. These strategies are based on our current understanding of interactions between the viral spikes and known neutralizing mAbs and incorporate lessons from studies with other viruses. Since more than 74,000 SARS-CoV-2 isolates already exist and since more can arise [13], structural information can be harnessed for modifying or improving existing vaccine designs and therapeutic strategies and for assessing the efficacy and quality of vaccine responses.

## 2. Structural Organization of the Spike

**Figure 1:**
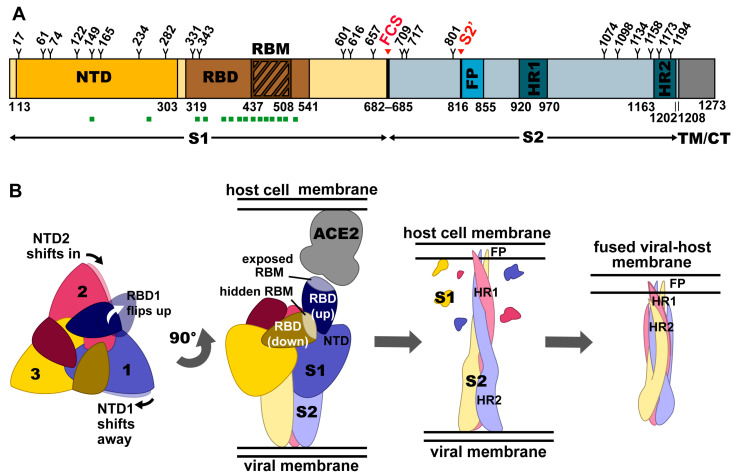
Structure and conformations of the SARS-CoV-2 trimeric spike protein. (**A**) Domain architecture of SARS-CoV-2 S, comprising the N-terminal domain (NTD), receptor-binding domain (RBD), receptor-binding motif (RBM), furin cleavage site (FCS), S2′, fusion peptide (FP), and heptad repeats 1 and 2 (HR1 and HR2), as they relate to the S1 and S2 subunits, as well as the transmembrane domain (TM) and cytoplasmic tail (CT). Glycosylation sites are numbered and marked with a Y. Regions of S that bind to known neutralizing monoclonal antibodies (mAbs) and nanobodies (VHHs) are indicated with green squares. (**B**) Conformational changes in the spike ectodomain during membrane fusion. Left: top view of prefusion SARS-CoV-2 S. Conformational changes in adjacent NTDs as the RBD of one protomer shifts into the up position are indicated with arrows. Middle-Left: Side view of prefusion SARS-CoV-2 S with 2 RBDs in the down conformation (RBMs hidden) and one RBD in the up conformation (RBM exposed), bound to the ACE2 receptor. Middle-Right: Side view of postfusion SARS-CoV-2 S with S1 shed and S2 subunits elongated towards the host cell membrane with FP inserted. Right: Side view of postfusion SARS-CoV-2 S after a collapse allowing HR2 to form a six-helix bundle with HR1, resulting in fusion of the viral membrane with the host cell membrane.

Like the spikes of SARS-CoV and MERS-CoV, the SARS-CoV-2 spike, S, is a trimeric class I fusion protein and can be divided into the receptor-binding S1 and the membrane-anchored S2 subunits (Figure 1A) [14,15,16]. Each S1 subunit contains an N-terminal domain (NTD) and a receptor-binding domain (RBD), sometimes referred to as the C-terminal domain (CTD). The RBD can be further subdivided into a fairly conserved core region and a more variable receptor-binding motif (RBM) [15]. The RBM of SARS-CoV-2 interacts with the host cell receptor, angiotensin-converting enzyme 2 (ACE2) (Figure 1B). SARS-CoV utilizes the same receptor [17,18], but MERS-CoV uses dipeptidyl peptidase 4 (DPP4) instead [19]. The S2 subunit contains the fusion machinery of the spike.

## 3. Interactions of the Spike with Its Receptor 

**Figure 2:**
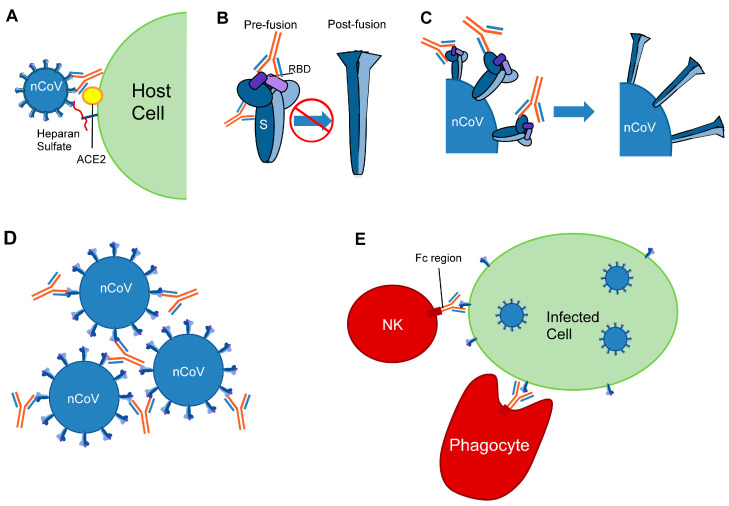
Mechanisms of antiviral protection by anti-coronavirus antibodies. Mechanisms include: (**A**) Inhibition of receptor binding: S interacts with heparan sulfate and ACE2 in the process of viral fusion. Antibodies obscure the ACE2 receptor-binding motif. (**B**) Antibody binding interferes with the prefusion to postfusion transition, preventing membrane fusion. The RBDs are shown in purple. (**C**) Antibody binding destabilizes the spike and leads to premature triggering of the postfusion conformation. (**D**) Antibodies bind multiple virions to form aggregates. (**E**) Fc-mediated functions include opsonization and activation of the complement system. Natural killer (NK) cell and phagocyte are shown.

S is conformationally dynamic and its RBD has been observed in the “up”, or open, and the “down”, or closed, states. More often, one S RBD exists in the up state [20]. S can only bind ACE2 (or DPP4 in the case of MERS-CoV) when the RBD is in the up state (Figure 1B) [16,21,22,23]. Heparan sulfate has been suggested to interact with the RBD to enhance its open state for ACE2 binding to occur (Figure 2A) [24]. On the other hand, the closed state is favored at endosomal pH (~5.5) [25]. S interacts with its receptor with high affinity, with both the SARS-CoV-2 S RBD and trimer binding to monomeric ACE2 with low nanomolar dissociation constants (K_D_) (subnanomolar in the case of dimeric ACE2) [15,22]. The K_D_ is lower than what is observed for the SARS-CoV RBD binding to ACE2 [21,22,26,27], although the full S trimeric ectodomains have similar affinities [14,26].

Multiple structures of S in complex with ACE2 have been determined [9,17,21,22,26,28] and both SARS-CoV-2 and SARS-CoV interact with the receptor in a similar manner [17]. Even though MERS-CoV S binds DPP4, it does so with a similar approach angle as observed between S from SARS-CoV and SARS-CoV-2 and ACE2 [19]. SARS-CoV 2 S and SARS-CoV S converge on a 17-residue ACE2 epitope on the RBM [22]. The RBM itself consists of a concave surface formed by extended loops stabilized by two β-hairpins. It is further stabilized by the RBD, which consists of a central β sheet flanked by several small alpha helices. The long N-terminal helix of ACE2 sits cradled on top of the RBM, and ACE2 residues K31 and K353 are critical for binding both SARS-CoV S and SARS-CoV-2 S [26,29]. S residues Y449, Q493, Q498, and N501 of SARS-CoV-2 contact these “hotspot” lysine residues, and when mutated, display altered affinity, highlighting their importance for S-ACE2 complex formation [30]. Interestingly, even though these S residues are important for ACE2 binding, there is degeneracy at these contact positions and so certain mutations can be tolerated. In fact, the SARS-CoV-2 S RBM sequence is only 47.8% identical to that of SARS-CoV S [31]. 

## 4. Viral Entry in Cells

In addition to depending on ACE2 for host cell entry, both SARS-CoV and SARS-CoV-2 depend on entry activation by host cell proteases at the S1/S2 and S2′ sites, regardless of whether entry occurs by fusion or endocytosis (Figure 1A) [10,32]. In contrast to SARS-CoV, however, SARS-CoV-2 also has a furin cleavage site that can be recognized by neuropilin-1 for infectivity [33,34]. Like other trimeric class I fusion proteins, S proteins from SARS-CoV, SARS-CoV-2, and MERS-CoV undergo dramatic structural changes to fuse membranes, which occurs after proteolytic activation at the S1/S2 boundary (Figure 1B) [35]. S1 dissociates, or “sheds”, and conformational changes allow the fusion peptide, which is rich in hydrophobic residues, to insert into the host cell membrane [15,36,37]. S2 forms an elongated structure, and the two heptad repeats, HR1 and HR2, eventually form a six-helix bundle to complete the fusion process and deliver the viral genome into the cytoplasm [16].

## 5. Viral Defense Mechanisms for Antibody Evasion

The importance of the viral spike to host cell entry makes it an ideal target for the immune system. SARS-CoV-2, however, evades the host immune response through conformational heterogeneity, glycosylation, mutation, and host peptide mimicry.

RBM-specific antibodies can sterically hinder ACE2 binding, making the RBM an ideal target for the immune system. The virus, however, effectively evades antibody binding to the RBM and certain parts of the RBD through conformational heterogeneity. S has been observed with either zero, one, two, or three RBDs in the up state, and the RBM is concealed when in the down state. In the case of SARS-CoV-2, the RBD is primarily in the down state [14], whereas in MERS- and SARS-CoV, it is primarily in the up state [23]. This evasion likely contributes to the high virulence of SARS-CoV-2. Moreover, while the RBD core structure is the same in both up and down states, small conformational changes do occur in other parts of the trimer. The NTD shifts slightly between RBD up and down states (Figure 1B) [38], and the conformation of the receptor-binding loop of SARS-CoV changes upon ACE2 binding [39]. However, the effects on antibody recognition through these changes are not yet well characterized. 

Glycosylation also plays a role in influencing the conformation of the RBD, as well as masking S from antibodies. There are 22 N-linked glycosylation sites (Figure 1A) along each SARS-CoV-2 S protomer (23 in the case of SARS-CoV) [40]. Some glycans are found near the receptor binding site, providing steric shielding from antibody binding and stabilizing the RBD in the up state [40,41,42,43,44]. S has a lower oligomannose-type glycan content (~30%) compared to spikes from the more evasive viruses HIV-1, influenza, and Lassa (>50%), leading to a less dense glycan shield and more accessibility to antibodies [41,45,46,47].

RNA viruses are prone to mutation, and variants of SARS-CoV, MERS-CoV, and SARS-CoV-2 have been identified [48]. Amino acid changes have been observed across the entire spike, including some glycosylation sites [44,49]. Several of these changes alter the binding of neutralizing mAbs to SARS-CoV-2, as well as SARS-CoV and MERS-CoV [44,50,51,52]. For example, the prevalent D614G variant of SARS-CoV-2 has shown resistance to a selection of neutralizing mAbs, including 4-fold increased EC_50_ to several mAbs targeting the RBM and RBD core regions of S [44,49]. This provides another means of immune evasion, although the mutation rates of coronaviruses are much lower than those of rapidly evolving pathogens such as HIV-1 and influenza [53].

The SARS coronaviruses also evade immune surveillance by mimicry of host peptides. For example, the SARS-CoV-2 S1/S2 cleavage site mimics an identical furin-cleavable peptide on the human epithelial sodium channel α-subunit [54]. Several peptides encoded by SARS-CoV-2 and SARS-CoV, including at the S2 domain, were also found to have high sequence homology with human proteins [55,56]. Moreover, these similarities have been found in human peptides known to bind MHC class-I molecules, identifying peptide mimicry as a mode of immune evasion by SARS-CoV-2 [57].

## 6. Anti-SARS-CoV-2 Neutralizing Epitopes

Despite viral defense mechanisms, mAbs and VHHs that target SARS-CoV-2 S have been identified and characterized. Most mAbs have been isolated from COVID-19 convalescent patients, though some derive from SARS-CoV patients or from transgenic mice. VHHs have been produced from immunization of llamas or alpacas or from synthetic libraries. Numerous three-dimensional structures of mAbs and VHHs in complex with S, either as a full trimeric ectodomain or as an isolated recombinant RBD, highlight immunodominant regions. Most mAbs against SARS-CoV-2 that have been structurally characterized target the RBM, although some RBD core- and NTD-specific mAbs have been characterized as well (Table 1). Comparisons with mAb epitopes on SARS-CoV and MERS-CoV S, as well as on spikes of unrelated viruses, provide insight into additional epitopes on SARS-CoV-2 that could be targeted by neutralizing mAbs. These potential new epitopes on S could inform the design of antigens to isolate mAbs against such sites that may offer broader protection, and the development of new combination therapies to prevent or overcome viral resistance. It is important to note that the most significant component of neutralizing epitopes are certain residues that make contacts with interacting mAbs. Even a single residue mutation may result in drastically different mAb neutralization potentials, despite the possibility of maintained binding affinities [58].

### 6.1. Receptor-Binding Domain: The Receptor-Binding Motif

MAbs that bind to the RBM block ACE2 binding and thereby prevent viral entry into host cells (Figure 2A). Structurally characterized RBM-binding mAbs fall into different functional classes, and some can be further separated into clusters based on epitope and approach angle.

#### 6.1.1. RBM Class I—Direct ACE2 Competitors That Bind Up RBD 

RBM Class I is the largest group of structurally characterized mAbs against the SARS-CoV-2 S RBM (Table 1). These mAbs have extensive overlap with the ACE2 epitope and can only bind when the RBD is in the up state since the ACE2 epitope is occluded in the down state. Moreover, IgG binding to the up state of the RBD can create a possibility for inter- or intra-spike crosslinking [63]. 

**Figure 3:**
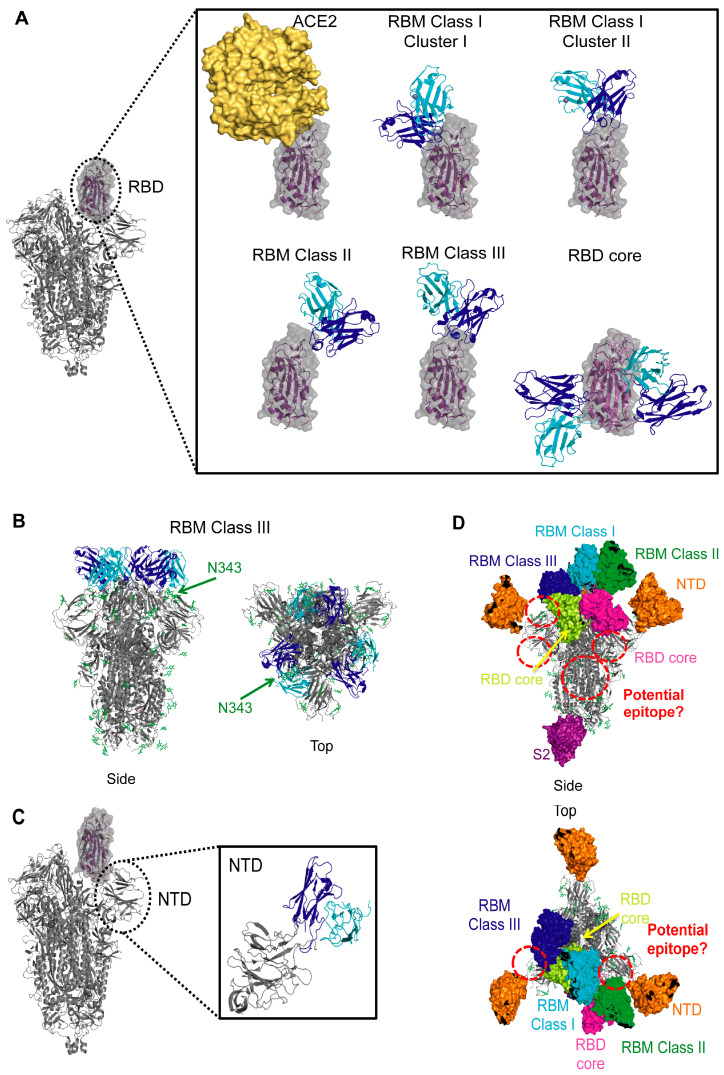
Coronavirus spike epitopes. (**A**) SARS-CoV-2 RBM epitopes. Side view of SARS-CoV-2 trimer (PDB ID 6X2A [20]) shown as gray cartoon with one RBD in the up state (shown as purple cartoon with gray surface). ACE2 (yellow surface, PDB ID: 6M0J [22]) or representatives of RBM-specific antibodies are shown bound to a zoomed in view of the RBD. Antibody variable heavy chain (blue) and light chain (cyan) are shown; constant domains are omitted for clarity. Representative members are shown for each class: CC12.3 from RBM Class I, Cluster I (PDB ID 6XC4 [59]), COVA2-39 from RBM Class I, Cluster II (PDB ID 7JMP [65]), P2B-2F6 from RBM Class II (PDB ID 7BWJ [72]), S2M11 from RBM Class III (PDB ID 7K43 [71]), and CR3022 (left) and S309 (right) from RBD Core (PDB IDs 6W41 [86] and PDB ID 6WPS [80]). (**B**) RBM Class III antibodies that lock the trimer in a “closed” state. Side and top views are shown for a representative antibody (S2M11) bound to the SARS-CoV-2 trimer (PDB ID: 7K43 [71]). Glycans are shown as green sticks and the location of an N343 from a single protomer is indicated. (**C**) SARS-CoV-2 NTD epitope. Side view of SARS-CoV-2 trimer (PDB ID 6X2A [20]) shown as gray cartoon with one RBD in the up state (shown as purple cartoon with gray surface). A zoomed in view of the NTD is shown with a representative NTD-specific antibody (4A8, PDB ID 7C2L [85]) bound. Antibody variable heavy chain (blue) and light chain (cyan) are shown; constant domains are omitted for clarity. (**D**) Epitopes of coronaviruses. Representative antibodies are shown as colored surface: cyan for CC12.3 from RBM Class I (PDB ID 6XC4 [59]), green for P2B-2F6 from RBM Class II (PDB ID 7BWJ [72]), blue for S2M11 from RBM Class III (PDB ID 7K43 [71]), yellow for CR3022 and magenta for S309 from RBD Core (PDB IDs 6W41 [86] and PDB ID 6WPS [80]), orange for 4A8 from NTD (PDB ID 7C2L [85]), and purple for G4 from anti-MERS-CoV S2, (PDB ID 5W9H [87]). Glycans are shown as green sticks. Potential epitopes are indicated with red circles.

A close look at the complexes with S of RBM-specific Class I mAbs, determined by X-ray crystallography or cryo-electron microscopy (cryo-EM), reveals that these mAbs, except for S2E12 [71], display two general binding modes, depending on the length of their CDRH3 loop (Figure 3A). Those with short CDRH3 (~7–11 amino acids) form the majority of Class I mAbs and all of these “Cluster I” mAbs bind with nearly identical approach angles and similar footprints on the RBM (Figure 3A). These include the following mAbs isolated from the sera of convalescent COVID-19 patients: B38 [66], CB6 [67], C102 [63], C105 [64], CC12.1 [59], CC12.3 [59], CV30 [60], COVA2-04 [61], BD236 [62], BD-604 [62], BD-629 [62], and a mAb isolated from a transgenic mouse: REGN10933 [68]. They use their heavy chain to interact with the RBD ridge and their light chain to interact with the flat surface of the ACE2 binding site. Most of these mAbs, except REGN10933, have IGHV3-53 or IGHV3-66 heavy chain gene usage. Of note, IGHV3-66 is identical to IGHV3-53 except for one amino acid substitution located in a non-CDR region [64]. Germline-encoded residues make key interactions with the RBM. In particular, these mAbs typically have “NY” and “SGGS” sequence motifs in their CDRH1 and CDRH2 loops, respectively, that are critical for binding the RBM. REGN10933 is an exception in that it only has the “NY” motif. These mAbs typically overlap with RBD sites that contact the K353 and K31 hotspots of ACE2. The heavy chains dominate interactions with S, explaining the similarity in their epitopes, despite having different light chain gene usages.

RBM-specific Class I Cluster II mAbs with long CDRH3 loops (at least 15 amino acids) bind to the RBM with a different binding mode in which the antibody fragment antigen-binding (Fab) domain is rotated 180 degrees along its long axis, swapping the relative orientations of the heavy and light chains (Figure 3A). In this case, the heavy chain interacts with the flat surface of the ACE2 binding site, and both heavy and light chains interact with the RBD ridge. MAbs COVA2-39 [65], COVA07-250 [70], and S2H14 [69], from the sera of COVID-19 convalescent patients, fall into this category and bind similarly to one another, angled more to the exterior of the trimer. Of note, COVA2-39 also uses IGHV3-53, illustrating this gene’s versatility in producing mAbs with different binding modes [65].

#### 6.1.2. RBM Class II—ACE2 Blockers That Bind Up and Down RBD

RBM-specific Class II neutralizing members consist of mAbs isolated from COVID-19 convalescent patients and VHHs isolated from immunizations of llamas or alpacas or from synthetic libraries (Table 1). Structures of their complexes have revealed that these mAbs and VHHs bind with an angle further from the center of the trimer, almost orthogonally relative to ACE2, allowing them to bind the RBD in the up or down states. Like Class I mAbs, neutralization can occur by preventing ACE2 from binding, though some Class II members may also neutralize the virus by inducing S1 shedding by trapping the RBDs in the up state (Figure 2C). This premature conversion to the postfusion state prevents viral–host membrane fusion.

RBM-specific Class II members (mAbs P2B-2F6 [72], BD-368-2 [62], CV07-270 [70], S2H13 [69], C002 [63], C104 [63], C119 [63], and C121 [63] and VHHs H11-D4 [73], H11-H4 [73], Ty1 [75], and Sb23 [74]) have epitopes that overlap with those of ACE2 even less than Class I members (Figure 3A). They recognize an epitope found within the crevice formed by the RBM β-hairpin, and many of them overlap with Y449, a residue also recognized by ACE2 [22]. MAbs C002, C104, C119, and C121 and VHHs H11-D4 and H11-H4 are exceptions and overlap with other ACE2 contacts, most notably E484 and/or Q493 [63,73]. Additionally, heavy or light chains of Class II mAbs clash with one side of ACE2 bound to the same RBD. Their constant domains could also potentially clash with dimeric ACE2. Moreover, when bound to an RBD in the down state, both mAbs and VHHs of this class clash with an ACE2 binding to a nearby RBD in the up state. Due to their proximity to a nearby RBD, these mAbs and VHHs could potentially stabilize the spike in a certain conformation and prevent “breathing” motions of the RBD. Despite their similar epitopes, no common variable region gene usage is observed among this class, although several of the VHHs use IGHV3-3.

#### 6.1.3. RBM Class III—ACE2 Blockers with Quaternary Epitopes

A prominent distinguishing feature between Class III RBM-directed mAbs and those of Class I and Class II is the interaction of these mAbs with RBDs of nearby protomers in the down state, potentially locking the spike in a closed conformation and preventing access to the ACE2 binding site (Figure 2B, Table 1). While our examination of these structures would suggest that these mAbs can bind to a single RBD in the up or down state, these interactions are not observed, indicating that elements from at least two RBDs in the down state are necessary for binding by each Fab. 

The structures of mAbs BD-23 [77], 2–4 [76], C144 [63], and S2M11 [71] and VHHs Nb6 [79] and Nb20 [78] in complex with the prefusion-stabilized SARS-CoV-2 S trimer show that they bind near the trimer apex, straddling the interface between two RBDs, and can potentially interact with the N343 glycan of the nearby protomer’s RBD (Figure 3A,B). The structure of S in complex with S2M11 showed that the N343 glycan was rotated by 45° relative to that in complex with S309, a non-RBM mAb, suggesting that contacts made with this glycan are significant [71]. Even though nanobodies have a smaller footprint, the Nb6 heavy chain interacts with a similar site on S as the heavy and light chains of the mAbs. Several of these mAbs, including BD-23 and 2–4, have also been suggested to interact with the N-linked glycan on the adjacent protomer’s NTD (N165). However, more biochemical work and neutralization studies with and without this glycan are needed to determine the effect it has on interaction with the RBD and the trimeric spike’s structural dynamics [76,77]. Despite their similar epitopes, there is diversity in the variable region gene usage among Class III RBM-directed members, all isolated from COVID-19 convalescent patients.

### 6.2. Receptor-Binding Domain: The Highly Conserved Core 

While the S RBM is an important target for SARS-CoV-2 neutralization because mAbs targeting this site compete with ACE2 binding, cross-neutralization with other coronaviruses is more likely to occur by mAbs that bind more conserved regions, such as the RBD core. MAbs of this class do not interact with the RBM and bind the RBD core in two general areas—one that is exposed when the RBD is down, and another site that is only accessible when the RBD is up (and, in some cases, rotated as well) (Table 1, Figure 3A). These mAbs can prevent ACE2 from binding using several different mechanisms: by directly clashing with ACE2 binding to the same RBD, by indirectly clashing with ACE2 on a nearby protomer, or by locking the trimer in a closed state, thereby concealing the ACE2-binding site. Some mAbs can even induce S1 shedding. Other neutralization mechanisms are also possible. 

MAbs C110 [63] and REGN10987 [68] bind with a similar pose to the RBD face accessible in both the open and closed states and can sterically hinder ACE2 binding. While mAbs 2–43 [76], S309 [80], and C135 [63] can also bind to the RBD in the down state, they do not compete with ACE2 for binding to the RBD (Figure 3A). Three copies of 2–43 bind to S and each Fab recognizes a quaternary epitope, requiring elements of two adjacent RBDs in the down conformation to bind [76]. This suggests that 2–43 can lock the spike in a closed conformation. Unlike 2–43, S309 and C135 can bind to the RBD in the open state [63,80]. Neutralization experiments comparing the effects of using an S309 Fab versus S309 IgG suggest that the IgG may induce neutralization by steric hindrance or via bivalent mechanisms, such as cross-linking S trimers or aggregating virions [80] (Figure 2D). The mechanism of neutralization by C135, a SARS-CoV-2-specific mAb, is not clear and requires further investigation [63]. Of these, only S309, which was actually isolated from a SARS-CoV patient, can neutralize both SARS-CoV-2 and SARS-CoV (Table 1).

On the opposite face of the RBD are binding epitopes for mAbs CR3022 [81,86], EY6A [82], S2X35 [69], S2A4 [69], S304 [69], and H014 [83], and nanobody VHH-72 [84] (Figure 3A). Of these, S2X35, S2A4, H014, and VHH-72 clash with ACE2. CR3022, EY6A, S2X35, S2A4, and S304 bind to a cryptic epitope that is only accessible when at least two RBDs are in the up state. In some cases, all three RBDs need to be up and/or rotated [81,86]. Of note, when bound to these mAbs, some of these RBDs swing out to unique (and unstable) states. Locking the trimer in this state can destabilize it and promote S1 shedding [81] (Figure 2C). The H014 mAb and VHH-72 nanobody have binding approaches in between those of CR3022 and S309 and they also require the RBD to be in the up state for binding, thereby locking it in that state. Several members of this cluster can neutralize both SARS-CoV-2 and SARS-CoV (Table 1). Of these, CR3022 and S304 were isolated from individuals infected with SARS-CoV (although CR3022 may not neutralize SARS-CoV-2 [86,88]), and H014 was isolated from a transgenic mouse.

### 6.3. NTD

Many studies that identified RBM- and RBD core-specific mAbs used the isolated recombinant RBD as bait. This approach would, however, neglect other immunogenic epitopes on the spike, such as the NTD. While the function of the NTD is not well understood, NTD-specific mAbs have been observed to attribute part of their neutralization potential to inhibiting the prefusion to postfusion conformational change of the spike after receptor binding occurs (Figure 2B, Table 1) [89]. Additionally, even though the NTD is located near the top of the trimer, far from the three-fold axis of the trimer and further out than the RBD, the MERS-CoV NTD-specific mAbs, 7D10 and G2, could still sterically interfere with receptor binding [89,90]. ACE2, a dimer, could also potentially clash with mAbs that bind the SARS-CoV or SARS-CoV-2 NTD. 

NTD-specific mAbs, such as 4A8 [85] and 4–8 [76] which were isolated from COVID-19 convalescent patients, can bind regardless of what state the RBD is in. MAbs 4–8 and 4A8 bind with almost identical angles to each other and to MERS-CoV mAbs 7D10 [89] and G2 [90], facing diagonally and upwards away from S2 (Figure 3C). These mAbs do interact differently with the NTD, however, due to differences in this subdomain when comparing these two coronaviruses. While both NTDs have flexible loops on the exterior of the central β-sandwich core, the MERS-CoV S also has several alpha helices on the NTD surface. Moreover, 4–8 and 4A8 bind further away from the central three-fold axis of the spike than the MERS-CoV mAbs and are less likely to interact with the receptor binding to the same spike, though steric interference might still be possible in the context of whole virions. These two antibodies have different gene usages when compared to each other and to antibodies from the other classes.

## 7. Other Potential Immunogenic SARS-CoV-2 Epitopes

Analyses of other coronavirus S mAbs, together with those against spikes of enveloped viruses which undergo fusion through similar mechanisms, could reveal new SARS-CoV-2 S epitopes against which mAbs have not yet been structurally characterized. Moreover, while none of the MERS-CoV mAbs investigated thus far have shown cross-reactivity with SARS-CoV-2 S, the investigation of unique MERS-CoV epitopes targeted by anti-MERS-CoV mAbs can still help identify new neutralizing epitopes on SARS-CoV-2 S. 

**Figure 4:**
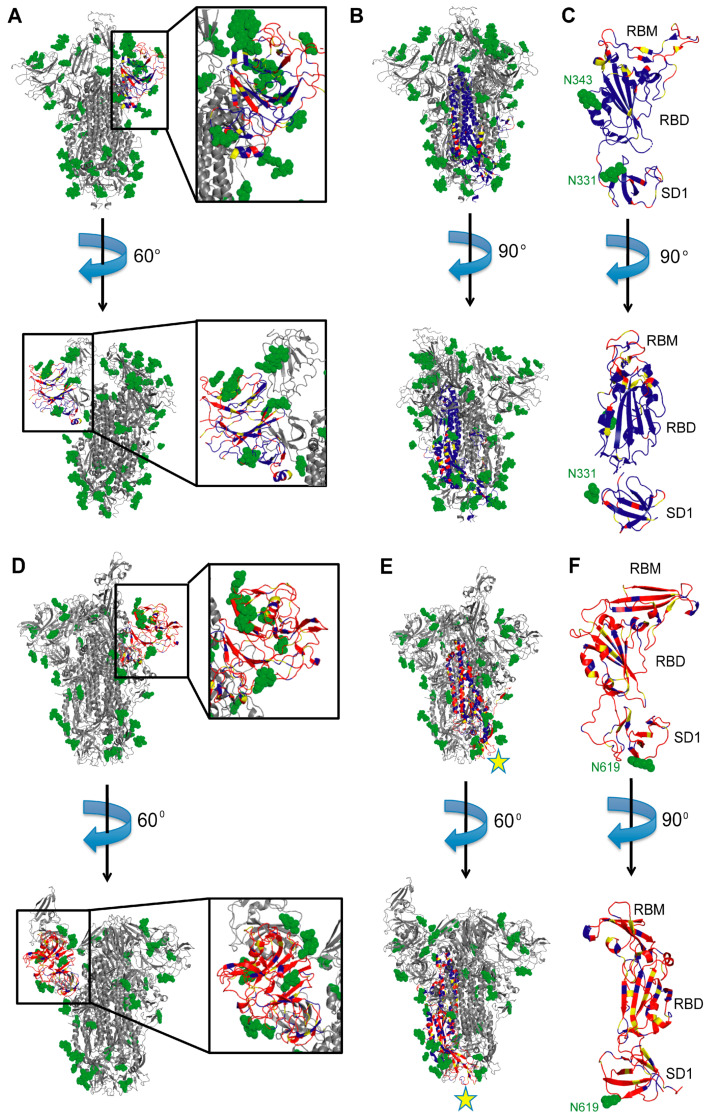
Surface conservation of S subdomains. (**A**) SARS-CoV-2 S NTD. Two side views of the S trimer are shown with the NTD zoomed in. Blue and red regions in the NTD of a single S protomer are conserved and variable, respectively, between SARS-CoV and SARS-CoV-2. Yellow regions are semi-conserved. Glycans are represented by green spheres. The rest of the trimer is in gray. PDB ID 7C2L used to generate the figure [85]. (**B**) SARS-CoV-2 S2 subunit. Two side views of the S trimer are shown. Coloring scheme is the same as in (**A**), with the S2 domain of a single protomer colored according to sequence conservation. PDB ID 7C2L used to generate the figure [85]. (**C**) SARS-CoV-2 S RBD. Two side views of the RBD and SD1 domains are shown with RBM at the top. Coloring scheme for sequence conservation is the same as in (**A**). PDB ID 7BZ5 used to generate the figure [66]. (**D**) MERS-CoV S NTD. Two side views of the S trimer are shown with the NTD zoomed in. Blue and red regions are conserved and variable, respectively, between MERS-CoV and SARS-CoV. Yellow regions are semi-conserved. Glycans are represented by green spheres. The rest of the trimer is in gray. PDB ID 5W9H used to generate the figure [87]. (**E**) MERS-CoV S2 subunit. Two side views of the S trimer are shown. Coloring scheme is the same as in (**D**), with the S2 domain of a single protomer colored according to sequence conservation. PDB ID 5W9H used to generate the figure [87]. mAb G4 binding site is indicated with a yellow star. (**F**) MERS-CoV S RBD. Two side views of the RBD and SD1 domains are shown with RBM at the top. Coloring scheme for sequence conservation is the same as in (**D**). PDB ID 5W9H used to generate the figure [87].

The MERS-CoV NTD-specific mAbs 7D10 [89] and G2 [90] interact with the spike similarly to those discussed above against SARS-CoV-2, in a region that is poorly conserved among the coronaviruses and therefore likely to be strain-specific (Figure 4A,D). Of note, antibodies from the COVID-19 patient COV57 [64] were also reported to bind to the NTD; however, this study utilized polyclonal electron microscopy, and so individual antibodies were not isolated for further characterization. In stark contrast to the NTD-specific mAbs mentioned above, NTD-specific antibodies from COV57 bound to the bottom face of the NTD, suggesting that other NTD regions could be immunogenic.

SARS-CoV-2 S2-specific mAbs have been isolated but have yet to be structurally characterized in the context of S2 or the full spike trimer. A MERS-CoV S2-specific mAb, called G4, has been structurally characterized [87]. G4 binds to a glycosylated, solvent-exposed variable loop emanating from two β-strands near the bottom of the S2 subunit, close to the viral membrane. This region is fairly conserved between SARS-CoV and SARS-CoV-2 but is very different in MERS-CoV (Figure 4B,E), suggesting that G4 is likely specific for MERS-CoV S. However, mAbs against this site of SARS-CoV-2 S might exist and even cross-neutralize SARS-CoV. There are also other S2 regions that are conserved across all three of these coronaviruses that may be immunogenic and worth studying (Figure 4B,E). Consistent with this, mAbs against analogous regions to S2 are found to target other class I trimeric fusion spikes, such as those of HIV and influenza. Several such mAbs were determined to be broadly neutralizing, targeting many viral variants [91,92,93]. Thus, S2-specific mAbs can prove to be very useful, and their binding could inhibit conformational changes necessary for membrane fusion to occur.

MAbs that bind to other S sites may also inhibit the conformational changes necessary for fusion. For example, in the case of HIV, broadly neutralizing mAbs have been identified against the V3 glycan supersite [94,95,96] and V1V2 loops of gp120 [97], a subunit analogous to the SARS-CoV-2 S1 subunit, as well as the gp120–gp41 interface [98,99], comparable to the area where S2 meets the RBD on SARS-CoV-2 S. MAbs against these sites generally prevent conformational changes that are required for binding a co-receptor or for fusion to occur (reviewed in [100]), suggesting that sites at the RBD–NTD, RBD–SD1, SD1–S2, or RBD–S2 (Figure 3D) interfaces might elicit neutralizing responses that have yet to be structurally characterized.

## 8. Epitope Conservation and Cross-Reactivity

MAbs against other coronaviruses hold potential as therapeutics against SARS-CoV-2 S and vice versa. Moreover, therapeutic administration of such mAbs would be approved more rapidly for distribution against new strains if they are already in use. Additionally, epitopes of cross-reactive mAbs hold promise in designing antigens to isolate broadly neutralizing mAbs as well as for designing immunogens to elicit such mAbs.

The RBD core, including the N343 glycan (N330 in SARS-CoV) with which several mAbs against this site must interact, is fairly conserved (Figure 4C) [79]. Moreover, some contact residues for RBD-directed cross-neutralizing mAbs are also conserved in other clade 1, 2, and 3 human and animal sarbecoviruses, suggesting possible cross-reactivity to other zoonotic sarbecoviruses. Thus, it is not surprising that several RBD core-specific mAbs, including EY6A, S304, S309, H014, and nanobody VHH-72, were demonstrated to be cross-reactive with SARS-CoV [69,80,82,83,84]. CR3022 was also shown to be cross-reactive in some studies, but not others [81,86,88]. The cryptic epitope targeted by several of these mAbs, particularly CR3022, EY6A, and S304, is likely important for stabilizing the closed conformation of the prefusion trimer, suggesting a low likelihood that resistance mutations would occur in this region. However, many of these mAbs are weakly neutralizing (Table 1), so their epitopes may not have therapeutic utility against SARS-CoV-2. The epitope on the opposite face of the RBD targeted by S309 and the epitope targeted by H014 are more promising [80,83]. Indeed, both S309 and H014 are in clinical trials (NCT04545060, NCT04483375, NCT04644185, and NCT04683328 in ClinicalTrials.gov).

While it has not been tested yet, the SARS-CoV RBD core-specific neutralizing mAb F26G19 could potentially target SARS-CoV-2 [101]. It has a highly conserved epitope (75% (6/8) residual sequence homology with SARS-CoV-2 S). Its binding is most similar to H014 and VHH-72, which cross-neutralize both of these viruses. F26G19 has an epitope and approach angle shifted more closely to the RBM and hence would have greater steric interference with ACE2 binding. Our modeling suggests it can only bind to RBD in the open state, since binding to a closed RBD would introduce steric interferences with a nearby up or down RBD. Thus, it may also neutralize the viruses by promoting S1 shedding.

Other sites on S are less conserved, so antibodies against certain epitopes are unlikely to be cross-reactive. Specifically, antibodies against the RBM, which is less than 50% conserved between SARS-CoV and SARS-CoV-2, are strain-specific; all of the RBM-specific Class I–III mAbs discussed above are specific for SARS-CoV-2 (Table 1). Even the SARS-CoV RBM-specific neutralizing mAb m396 cannot bind SARS-CoV-2 S, despite its epitope having 77% homology with that of SARS-CoV [102]. This could be explained by the fact that only a small fraction of mutations could eliminate binding. Other mAbs against the RBM have yet to be tested for cross-reactivity, including the anti-SARS-CoV mAb 80R (whose epitope is only 45% (13/29) homologous with the corresponding SARS-CoV-2 epitope) [103]. The RBM of MERS-CoV S is different structurally, with a β-sheet replacing one of the β-hairpins found in S of SARS-CoV and SARS-CoV-2 (Figure 4C,F). Thus, MERS-CoV RBM-specific mAbs are less likely to be cross-reactive. Moreover, viral variants that emerge still retain binding to ACE2, suggesting that the RBM is likely to remain a strain-specific epitope.

NTD-specific and S2-specific mAbs that have been structurally characterized to date are also strain-specific. While the NTDs of SARS-CoV and SARS-CoV-2 S have a lower sequence identity (51%) than the RBDs (74%) [104], there are solvent-exposed regions of higher conservation, particularly at the bottom face of the NTD, possibly targeted by antibodies from patient COV57 (Figure 4A) [64]. While those antibodies were characterized by nsEM, higher-resolution data would be needed to provide insights into their ability to cross-neutralize SARS-CoV and SARS-CoV-2. In contrast to the NTD, S2 has a higher percentage of sequence identity among coronaviruses (Figure 4B,E), suggesting that mAbs against this region may be more effective in targeting a greater range of coronaviruses [104]. It is possible, however, that S2-specific mAbs are weakly or non-neutralizing. Their structural characterization will provide more insights into their neutralization potential and indicate whether S2 should be used to elicit and identify S2-specific mAbs.

## 9. Synergy and Overcoming Resistance Mutations 

Despite the sequence conservation in some areas of S, particularly in the RBD and S2 subdomains, resistance mutations can still occur in these regions. Specifically, consistent exposure to a single neutralizing mAb has been observed to induce viral escape mutations [105,106]. Moreover, point mutations in a neutralizing epitope can differentially influence the potency of different neutralizing mAbs, even if their epitopes overwhelmingly overlap. Greaney et al. developed a deep mutational scanning method to produce viral escape maps induced by specific mAbs [58]. In contrast to antibody–spike complex structures on their own, these maps can showcase which specific immunogenic residues are important for neutralization. These maps, together with high-resolution structures, provide a greater understanding of how frequent SARS-CoV-2 escape mutations have emerged [107]. Additionally, visualizing residual escape mutations directly informs the development of antibody cocktails consisting of mAbs that target unique residues, even in the case of overlapping epitopes [58]. Antibody cocktails that are administered simultaneously can overcome viral resistance and could also be more effective at virus neutralization than the individual mAbs alone. Even weak neutralizers used in combination with other mAbs could lead to potent neutralization [52,80]. 

Several studies have illustrated the promise of combining multiple mAbs. For example, a cocktail of REGN10933 (RBM-specific Class I) and REGN10987 (RBD core-specific) could prevent escape mutations from evading both mAbs [106]. BD-629 (RBM-specific Class I) was also shown to efficiently neutralize several naturally occurring S mutants that resist BD-368-2 (RBM-specific Class II) alone [62]. Synergistic neutralization was observed for S2E12 (RBM-specific Class I) and S2M11 (RBM-specific Class III) combined separately with S309 (RBD core-specific, solvent-exposed epitope) and for H11-H4-Fc (RBM-specific Class II) combined with CR3022 (RBD core-specific, cryptic epitope) [71,81]. MAbs that target opposite faces of the RBD were also shown to act synergistically. In particular, a combination of S309 with S304 was observed to enhance neutralization [80]. Additional mAbs, whose complex structures with S were not determined at high-resolution, have also shown synergistic effects. For example, COV2-2196 (RBM-specific) and COV2-2130 (RBD core-specific), which can bind to S simultaneously, synergize in vitro and their cocktail is protective in mouse models [105].

In contrast to RBM-specific and RBD core-specific mAbs, no NTD-specific mAbs, whose complex structures have been analyzed, have yet been tested for synergistic effects. NTD-specific mAbs would be good candidates for antibody cocktails, as their epitopes exhibit no overlap with those of the other antibody classes and therefore would cover a larger surface area on the spike when combined with RBD core-specific mAbs, further reducing the chance for resistance mutations to occur. The same would be true of certain S2-specific mAbs, should they be characterized. Finally, other antibody combinations besides those mentioned above, should be feasible in cocktails as well.

## 10. Multivalent and Multi-Specific Antibodies

MAbs and cross-linked nanobodies against certain sites on S can lead to more potent anti-viral effects than Fabs or nanobodies. For example, neutralization experiments with S309 Fab and IgG demonstrated that the latter could lead to 100% neutralization, whereas the Fab could not. This result, together with that from binding measurements showing that the IgG has a dissociation constant an order of magnitude smaller than that of the Fab, suggests IgG-specific bivalent mechanisms were at play [80], although affinity could be important as well. Barnes et al. also provided information on which mAbs could potentially be involved in inter- and intra-spike cross-linking [63]. What could use more attention, however, is the generation of multi-specific antibodies by combining fragments from different mAbs and/or nanobodies to accomplish specific antiviral actions, which have the potential to be more effective than certain cocktails alone. 

Bivalent interactions of IgGs or Fc–nanobody fusions would allow these constructs to interact with and aggregate different virions, cross-link different spikes within a virion, or provide greater steric hindrance, thereby contributing to neutralization. Thus, most nanobodies, when connected by a linker and fused to the Fc domain of human IgG1, displayed an increase in neutralization potency against SARS-CoV-2 pseudotyped viruses, with similar IC_50_ values as mAb IgGs [73,75,85,108]. These increases in neutralization were significant for some of the nanobodies, such as Sb23 (RBM-specific Class II), which had ~100-fold improved neutralization as an Fc-fusion, bringing its IC_50_ down to 7 ng/mL [74]. Generating a trivalent construct was demonstrated to have even greater effects in the case of Nb6 (RBM-specific Class III). Nb6 as a trivalent construct showed 2000-fold neutralization enhancement, bringing its IC_50_ down to 1.2 nM [79]. Of note, this enhancement could not be recapitulated for other nanobodies, suggesting the particular binding mode of Nb6 is important.

Bispecific antibodies have shown some promise against ebolaviruses, which enter host cells through endosomes, and HIV [109,110]. Thus, certain S mAbs and nanobodies might be useful for engineering bispecific antibodies against SARS-CoV-2 and other coronaviruses. Of note, it has been shown that at lower pH, representative of what is observed in the endosome, S is predominantly in the closed state [25]. As a result, mAbs that bind to a cryptic epitope or require the RBD to be in the up state would be less likely to bind and neutralize viruses fusing via the endosomal pathway. MAbs that bind the closed state, such as S309 (RBD core-specific) and S2H13 (RBM-specific Class II), were shown to be unaffected at lower pH [69]. Thus, the generation of a bispecific antibody from a combination of such a mAb, together with one that targets another site, could potentially exert its effects in the endosome upon viral recruitment. Such bispecifics, as well as others, might lead to enhanced neutralization breadth relative to their parental mAbs provided individually or in combination, similar to what was observed in the case of ebolaviruses [109]. However, rational design of effective bispecific antibodies is complex and certain mAbs used in cocktails might be more efficient than bispecifics, so investigations into the latter should be prioritized for therapeutic development. 

We do note that predictions based on static Fab/nanobody complex structures with S may not be sufficient for predicting which sites would serve as optimal targets because the spike and immunoglobulins are dynamic. Moreover, steric hindrances from immunoglobulin structures, particularly from the Fc region, are not apparent from available structural data. Thus, Fabs or nanobodies that appear to be able to bind simultaneously to the spike may not do so in vivo. A better understanding of the spike density on coronavirus virions and of how many spikes are necessary for fusion to occur would also give insights into how successful such strategies might be.

## 11. In Vivo Effects of mAbs 

In vivo neutralization would be a better predictor for the effectiveness of mAbs since in vitro experiments utilize systems with different numbers of spikes. Additionally, in vivo studies can provide insight into antibody effector functions involving their Fc domains, which are crucial for antiviral pathways and viral clearance. Such functions include antibody-dependent, cell-mediated phagocytosis (ADCP) of virions and infected cells, the direct killing of infected cells through antibody-dependent, cellular cytoxicity (ADCC), and the activation of the complement (Figure 2E). These effects rely on antibody Fc domains interacting with Fc gamma receptors (FcγRs), found on myeloid and natural killer cells. Some studies have used animal models to demonstrate the efficacy of SARS-CoV-2 S-targeting mAbs in vivo, and others have used cell-based assays to investigate effector functions of mAbs. Of note, no animal model has been found to reliably mimic COVID-19 disease progression in humans, and such studies should be regarded with an understanding that results may not perfectly reflect effects on humans. Nevertheless, studying the effects of mAbs on viral infection in complex living systems provides insights that in vitro studies alone cannot. 

When tested in human ACE2 transgenic mice, B38 (RBM-specific Class I) proved effective post-infection [66], and H014 (RBD core-specific) and BD-368-2 (RBM-specific Class II) were found to be effective both prophylactically and therapeutically [62,77,83]. BD-368-2 was also tested in hamsters and was found to offer treatment against SARS-CoV-2 challenge. CC12.1 (RBM-specific Class I), S2E12 (RBM-specific Class I), and S2M11 (RBM-specific Class II) also protected hamsters against viral challenge, and S2M11 even showed ADCC and ADCP activity [71,111]. ADCC was also observed with S309 (RBD core-specific) and S2H13 (RBM-specific Class II), though these studies were performed using cell-based assays [69]. Finally, while none of the nanobodies characterized at high resolution were tested in animals, an ACE2-competitive (likely RBM-specific) nanobody, Ab8, was fused with Fc and shown to be effective in mouse SARS-CoV-2 challenge studies and in both prophylactic and treatment regimens in a hamster model [108]. Thus, nanobodies can also work in animals, but further investigation is still needed. Further investigation is also needed for NTD-specific and RBM-specific Class III mAbs.

Several concerns arise when administering or eliciting mAbs. One is of mAb autoreactivity or polyreactivity. Another potential concern is that of antibody-dependent enhancement (ADE), a phenomenon where poorly-neutralizing antibodies aid viral entry into certain immune cells and can lead to more severe illness [112,113,114]. To test for autoreactivity, Kreye et al. investigated CV07-270 (RBM-specific Class II) and found that it reacted to murine smooth muscle from the lungs, heart, kidneys, and colon, but not the liver [70]. Several modifications have been suggested to prevent certain forms of autoreactivity. For instance, to prevent ADE, a LALA mutation was introduced into the Fc region of CB6 (RBM-specific Class I) to prevent Fc binding to an FcγR, and this mAb mutant was found to be effective when administered prophylactically and post-infection in rhesus macaques [67]. Native BD-368-2 (RBM-specific Class II) did not cause ADE, illustrating that these effects can vary and should be tested for individual mAbs [62]. Of note, current evidence of ADE in coronaviruses is limited to cell-based assays and animal models, and little evidence supports the notion of ADE actually occurring in humans (reviewed in [115]).

Early experimentation with administering convalescent patient plasma to severely ill COVID-19 patients has led to beneficial outcomes [2,5], inspiring hope that administration of mAbs might be a reliable treatment method for prevention and treatment of severe cases of COVID-19. The limited availability of plasma prevents its use as a widespread treatment, whereas mAbs can be produced on a large scale [77]. Clinical trials in humans are currently underway for several mAbs targeting SARS-CoV-2, including S309 (NCT04545060), BD-368-2 (NCT04551898, NCT04669262, NCT04532294), CB6 (NCT04441918), H014 (NCT04483375, NCT04644185, NCT04683328), REGN10933 in combination with REGN10987 (NCT04519437, NCT04426695, NCT04425629, NCT04452318, NCT04617535), COV2-2196 in combination with COV2-2381 (NCT04625972, NCT04625725), and LY-CoV555 (NCT04411628, NCT04427501, NCT04537910) and LY-CoV016 (NCT04441931), both alone and in combination. While few mAbs have been approved for widespread clinical use in the treatment of viral infections, numerous studies have shown antibodies to effectively protect against viruses, such as respiratory syncytial virus (RSV), HIV, and Ebola, which are in human clinical trials [116,117,118]. Studies with Fc mutations suggest that mAbs used for treatment of COVID-19 could be engineered in such a way to avoid the risk of ADE [113,119]. Optimizing interactions between Fc domains and specific FcγRs might further improve in vivo protection by mAbs, although further studies will be needed to determine the impacts of such modifications when used in humans.

## 12. Conclusions and Future Perspectives

The isolation of antibodies from convalescent individuals, together with their structural characterization through cryo-EM and X-ray crystallography, has accelerated the process of identifying features of SARS-CoV-2 S necessary for neutralization by mAbs and for eliciting such mAbs. Additional technologies, including the use of synthetic libraries, have helped identify neutralizing nanobodies that can be more rapidly generated against a desired antigen than those from llama or alpaca immunizations. It still remains to be determined how effective such nanobodies are in vivo. Moreover, humanized mouse platforms have shown promise in that S mAbs can be produced that bind in a similar way to those produced in humans. MAb REGN10933 represents such an example [68]. Further engineering of both nanobodies and mAbs to increase multivalency and/or improve effector functions may also improve therapies until the COVID-19 vaccine becomes widely accessible. In any case, a thorough understanding of critical residues for neutralization on various regions of SARS-CoV-2 S is crucial for vaccine design as well as for the selection of antibodies for passive administration.

Many of the mAbs produced against SARS-CoV-2 S have low rates of somatic hypermutations and use a diverse set of variable domain genes, demonstrating promise that similar mAbs can be produced rapidly via vaccination. Consistent with this, the BioNTech/Pfizer and Moderna vaccines, which produce different spike-related constructs, have shown to offer protection, even after the first of two doses [120,121]. Nevertheless, longitudinal analyses of antibody responses are still necessary to determine the long-term efficacy of these vaccine-induced antibodies against any potential viral variants that may arise. The notion of conferring protection against SARS-CoV-2 mutants raises the question of the possibility of a universal coronavirus vaccine that protects against other betacoronaviruses. Additional analyses would need to be done to address this, and the development of a coronavirus vaccine is a critical first step. Due to sequence and structural differences between various coronavirus spikes, other vaccine design strategies would need to be employed to design a universal vaccine. It may be the case that seasonal vaccines, such as those for influenza, may become necessary until such a universal vaccine is available, should other coronaviruses spread as much as the current one.

In the absence of a safe, effective, and widely available vaccine, passive antibody administration is an appealing alternative, despite likely having short-term beneficial effects. Escape mutations are a concern with passive administration of antibodies [106], but mAb cocktails and multi-specific antibodies targeting multiple distinct regions on SARS-CoV-2 S may eliminate this concern and offer synergistic effects. Additionally, careful selection for strongly neutralizing mAbs, along with engineered mutations in the Fc region, may reduce the risk of ADE, if it were to occur in humans. Nevertheless, with any potential antibody treatment, rigorous testing in clinical trials must be pursued. 

## Figures and Tables

**Table 1:** SARS-CoV-2 mAbs and VHHs whose high-resolution structures have been determined.

Epitope	Abs/Nbs and IDs *	Heavy V Gene	Light V Gene	IC_50_^#^/IC_50_^##^ (ng/mL)	Characteristics	Refs
RBM Class I	CC12.3 (6XC4)	IGHV3-53	IGKV3-20	18/26	Epitope directly overlaps that of ACE2. MAb binding mode also mimics ACE2 binding, requiring RBD to be in the up state. All members are strain-specific and many have IGHV3-53 or IGHV3-66 heavy chain gene usage with a variety of light chains.	[59]
	CV30 (6XE1)	IGHV3-53	IGKV3-20	30/118		[60]
	COVA2-04 (7JMO)	IGHV3-53	IGKV3-20	220/2500		[61]
	BD-629 (7CH5)	IGHV3-53	IGKV3-20	6/–		[62]
	C102 (7K8M)	IGHV3-53	IGKV3-20	34/–		[63]
	C105 (6XCM)	IGHV3-53	IGLV2-8	26.1/–		[64]
	COVA2-39 (7JMP)	IGHV3-53	IGLV2-23	36/54		[65]
	CC12.1 (6XC2)	IGHV3-53	IGKV1-9	19/120		[59]
	BD-604 (7CH4)	IGHV3-53	IGKV1-9	5/–		[62]
	BD-236 (7CHB)	IGHV3-53	IGKV1-9	37/–		[62]
	B38 (7BZ5)	IGHV3-66	IGKV1-9	–/177		[66]
	CB6 (7C01)	IGHV3-66	IGKV1-39	23/36		[67]
	REGN10933 (6XDG)	IGHV3-11	IGKV1D-33	6.4/5.6		[68]
	S2H14 (7JX3)	IGHV3-15	IGLV6-57	900/–		[69]
	CV07-250 (6XKQ)	IGHV1-18	IGLV2-8	–/3.5		[70]
	S2E12 (7K4N)	IGHV1-58	IGKV3-20	2.3/4.2		[71]
RBM Class II	P2B-2F6 (7BWJ)	IGHV4-38-2	IGLV2-8	50/410	Epitope directly overlaps that of ACE2, but less so than Class I members so they can bind RBD that is up or down. Clashes with ACE2 can also occur. Gene usage varies among this class. All members are strain-specific.	[72]
	BD-368-2 (7CHC)	IGHV3-23	IGKV2-28	1.2/15		[62]
	CV07-270 ** (6XKP)	IGHV3-11	IGLV2-14	–/82.3		[70]
	S2H13 (7JV2)	IGHV3-7	IGLV7-46	500/–		[69]
	C002 (7K8S)	IGVH3-30	IGVK1-39	8.9/–		[63]
	C104 (7K8U)	IGHV1-46	IGLV2-14	23.3/–		[63]
	C119 (7K8W)	IGHV4-34	IGKV3-20	9.1/–		[63]
	C121 (7K8X)	IGHV1-2	IGLV2-23	6.7/1.6		[63]
	H11–D4 (6YZ5)	IGHV3-3	NA	900 ^^^/–		[73]
	H11–H4 (6ZHD)	IGHV3-3	NA	300 ^^^/–		[73]
	Sb23 (7A29)	IGHV3-3	NA	600/–		[74]
	Ty1 (6ZXN)	IGHV3-48	NA	770/–		[75]
RBM Class III	2–4 (6XEY)	IGHV1-2	IGLV2-8	394/57	Similar properties as Class II, except these antibodies make contact with nearby RBDs in addition to the one(s) they are bound to. This limits conformational motions and some of them even lock the trimer in a closed state. Gene usage varies among this class. All members are strain-specific.	[76]
	S2M11 (7K43)	IGHV1-2	IGKV3-20	2.1/1.2		[71]
	C144 (7K90)	IGHV3-53	IGLV2-14	6.9/2.6		[63]
	BD-23 (7BYR)	IGHV7-4-1	IGKV1-5	4800/8500		[77]
	Nb20 (7JWB)	IGHV3-66	NA	1.5/0.7		[78]
	Nb6 (7KKK)	IGHV3S53	NA	30,000/15 ^$^		[79]
RBD Core, Cluster I	S309 *** (6WPS)	IGHV1-18	IGKV3-20	190/79	Prevent ACE2 from binding by either clashing with ACE2 or locking the trimer in a closed conformation. Gene usage varies among this class. S309 can cross-neutralize SARS-CoV and SARS-CoV-2.	[80]
	C135 (7K8Z)	IGHV3-30	IGKV1-5	16.6/3		[63]
	C110 (7K8V)	VH5-51	VK1-5	18.4/–		[63]
	REGN10987 (6XDG)	IGHV3-30	IGLV2-14	6.1/6.3		[68]
	2–43 (22,275)	IGHV1-2	IGLV2-14	71/3		[76]
RBD Core, Cluster II	CR3022 *** (6W41)	IGHV5-51	IGKV4-1	–/114 ^†^	Bind a cryptic epitope that is accessible only when the RBD is up and, in some cases, open as well. These members are capable of disrupting the trimer and promoting S1 shedding. Gene usage varies among this class. CR3022 ^†^, EY6A, S304, H014, VHH-72 can cross-neutralize SARS-CoV and SARS-CoV-2.	[81]
	EY6A (6ZCZ)	IGHV3-30-3	IGKV1-39	–/70–20,000		[82]
	S2A4 (7JVC)	IGHV3-7	IGLV2-23	3500/–		[69]
	S304 *** (7JW0)	IGHV3-13	IGKV1-39	>5000/–		[69]
	H014 *** (7CAH)	IGHV1-69-2	IGKV6-21	450/5700		[83]
	S2X35 (7JXE)	IGHV1-18	IGLV1-40	500/–		[69]
	VHH-72 *** (6WAQ)	IGHV3-3	N/A	200 ^^^/–		[84]
NTD	4A8 (7C2L)	IGHV1-24	IGKV2-24	49,000/390	MAbs of this class that have been structurally characterized thus far are strain-specific. Neutralization mechanism is unknown but may occur by preventing conformational changes necessary for fusion, or by steric interference with ACE2 on virions.	[85]
	4–8 (22,158)	IGHV1-69	IGLV3-1	32/9		[76]

* Structural ID provided (PDB ID or EMDB ID); ** autoreactive; *** cross-reactive with SARS-CoV; ^#^ IC_50_ from pseudovirus assay; ^##^ IC_50_ from assay using live virus; ^^^ Fc fusion was used; ^$^ trimerized nanobody was used; ^†^ conflicting neutralization reports across studies. Dashes indicate data are not available.

## Data Availability

Publicly available datasets were analyzed in this study. The data can be found in the Protein and Electron Microscopy Data Bank (PDB and EMDB) archives using PDB or EMDB IDs displayed in Table 1.
